# Safety and Efficacy of Orbital Atherectomy in the All-Comer Population: Mid-Term Results of the Lower Silesian Orbital Atherectomy Registry (LOAR)

**DOI:** 10.3390/jcm12185842

**Published:** 2023-09-08

**Authors:** Piotr Rola, Szymon Włodarczak, Mateusz Barycki, Łukasz Furtan, Artur Jastrzębski, Michalina Kędzierska, Adrian Doroszko, Maciej Lesiak, Adrian Włodarczak

**Affiliations:** 1 Faculty of Health Sciences and Physical Culture, Witelon Collegium State University, 59-220 Legnica, Poland; wlodarczak.adrian@gmail.com; 2 Department of Cardiology, Provincial Specialized Hospital, 59-220 Legnica, Poland; mateusz.barycki@gmail.com (M.B.); lukas.furtan@gmail.com (Ł.F.); 3 Department of Cardiology, The Copper Health Centre (MCZ), 59-300 Lubin, Poland; wlodarczak.szy@gmail.com (S.W.); art.jastrzebski@gmail.com (A.J.); 4 Faculty of Medicine, Wroclaw Medical University, 50-556 Wroclaw, Poland; kedzierska.michalinaa@gmail.com; 5 Department of Cardiology, Center for Heart Diseases, 4th Military Hospital, Faculty of Medicine, Wroclaw University of Science and Technology, 50-981 Wroclaw, Poland; adrian.doroszko@gmail.com; 6 1st Department of Cardiology, University of Medical Sciences, 61-848 Poznan, Poland; maciej.lesiak@skpp.edu.pl

**Keywords:** calcified lesion, orbital atherectomy, percutaneous coronary intervention, coronary artery calcifications, lesion preparation, mid-term outcomes

## Abstract

Background: Coronary calcifications represent a challenging subset for the interventional cardiologist performing percutaneous coronary intervention (PCI) and are well-established risk factors for adverse outcomes. Adequate plaque modification prior to stent implantation is critical to achieve an optimal outcome following PCI. Recently, a novel orbital atherectomy device has been introduced into clinical practice to modify calcified plaques. We evaluated the mid-term safety and efficacy of OA in a high-risk “all-comers” population. Methods: We evaluated 96 consecutive patients with severely calcified coronary lesions who underwent PCI facilitated by the orbital atherectomy device. Results: In-hospital MACCE was 5.2% without target lesion revascularization. At 6-month follow-up, the MACCE rate was 10.4% with a concomitant TLR rate of 1%. Conclusions: Our mid-term data showed good safety and efficacy of orbital atherectomy as a plaque-modifying tool in an all-comers cohort with severely calcified coronary lesions.

## 1. Introduction

Even up to one-third of patients undergoing percutaneous coronary intervention (PCI) have target lesions with moderate or severe calcification [1,2]. Severe coronary artery calcification (CAC) is a well-established risk factor for adverse outcomes in patients with coronary artery disease (CAD). Unreliable CAC complicates percutaneous coronary intervention (PCI) mainly by inhibiting optimal stent expansion [3,4], which is clinically reflected in an increased risk of death, myocardial infarction, repeat revascularization, stent thrombosis and restenosis [5,6,7].

In addition, patients with coronary calcifications undergoing PCI are less likely to receive complete revascularization, which has a strong impact on all-cause mortality [8,9,10]. Adequate lesion preparation prior to stent implantation to facilitate stent delivery and allow for optimal stent expansion is critical in the management of adverse events [11]. In contemporary practice, several dedicated devices have been used in order to achieve this goal. One of the most established devices with well-documented safety and efficiency is the rotational atherectomy device [12,13] (Boston Scientific, Marlborough, MA, USA). The main mechanism of action is focused on the ablation of calcified atherosclerotic coronary plaque by advancing a high-speed diamond-encrusted elliptical burr into the vessel. Despite its relatively high efficacy, RA has several shortcomings, mainly related to potential complications (dissection, perforation, microvascular obstruction, and burr formation). In addition, RA-assisted PCI is a rather demanding procedure with a “long” learning curve. Recently, a novel device based on the concept of atheroablation has been introduced into clinical practice. The orbital atherectomy device (Diamondback 360) (Cardiovascular Systems, Inc., St. Paul, MN, USA) is also a debulky device, yet it has a unique mechanism of action compared to RA. Briefly, it is a drive shaft with an eccentrically mounted diamond-coated crown that provides proximal and distal grinding to modify the plaque and increase the luminal size and compliance [14]. Despite OA already receiving FDA approval and the CE mark for Europe, data from “real-life” studies are still missing. Therefore, we designed this study to evaluate the mid-term safety and efficiency of OA in calcified coronary artery lesions.

## 2. Materials and Methods

### 2.1. Study Population

The study population consisted of 96 consecutive patients with severely calcified coronary lesions who underwent PCI facilitated by the orbital atherectomy device between May 2022 and January 2023. The present study is a retrospective, unblinded, single-arm study conducted in two cooperative high-volume centers. The study conforms to good clinical practice and the standards of a local ethics board.

Severe calcification was defined as radio-opacities seen without cardiac motion before contrast injection, involving both sides of the arterial wall, and usually occupying more than 50% of the reference diameter; or the presence of at least 2 points for the Intravascular Ultrasound-Derived Calcium Score [15,16,17].

### 2.2. PCI Procedure

All PCI procedures were performed by experienced PCI operators who completed a training program in orbital atherectomy and obtained the appropriate certificate.

**Figure 1:**
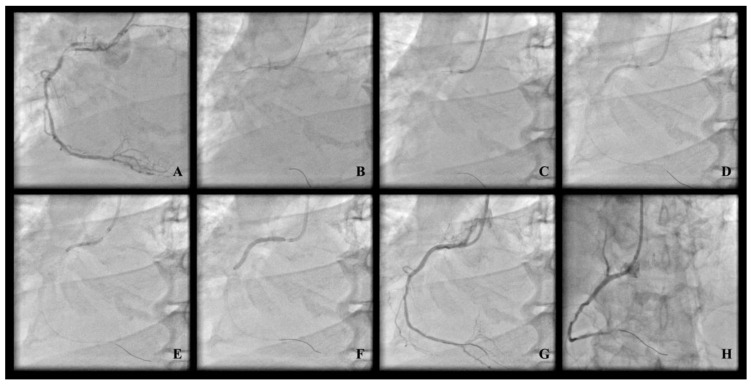
Exemplary OA-PCI. (**A**) Proximal calcified “uncrossable” lesion; (**B**) orbital atherectomy crown engagement; (**C**) reverse orbital atherectomy ablation; (**D**) NC balloon predilatation; (**E**) NC balloon predilatation; (**F**) DES implantation; (**G**) final angiographic result; (**H**) final angiographic result.

All procedural features (vascular access point, use of additional lesion preparation technique, stent implantation parameters, periprocedural pharmacological therapy, use of intravascular imaging support along with the left ventricular assist device) were left to the discretion of the operator. All OA procedures were started at OA at low speed (80,000 RPM) with additional passes at high speed (120,000 RPM) left to the discretion of the operator with strong encouragement to use high-speed mode only for a vessel diameter of at least 3.0 mm assessed based on coronary angiography. Despite prior stent implantation in the area of the target lesion, there were no other exclusion criteria regarding lesion anatomy (length, tortuosity, severity, or location). Figure 1 shows exemplary OA-PCI.

### 2.3. Study Device

The orbital atherectomy device (Diamondback 360) (Cardiovascular Systems, Inc., St. Paul, MN, USA) is a debulking device with an eccentrically mounted diamond-coated crown (1.25 mm). The device uses centrifugal force to orbit, allowing for atheroablation by grinding and abrasion of calcified plaque. This orbital movement of the crown allows blood and micro debris (smaller than the diameter of a red blood cell) to flow past the crown. In addition, to reduce thermal injury during the OA procedure, continuous infusion of ViperSlide fluid is performed. The OA Crown has the capability of bidirectional passage through the dedicated guidewire—0.012″/0.014″ tip ViperWire. In contrast to classical rotational atherectomy, there is only one size of crown without the ability to escalate the diameter; during the procedure, the operator can increase the lumen gain by increasing the time of contact with the lesion, the number of passes or the speed of rotation.

### 2.4. Study Outcomes

The study had two primary endpoints—clinical success and safety. Clinical success was defined as effective stent deployment without significant stent underexpansion [18] and the presence of thrombolysis in myocardial infarction (TIMI) 3 flow at the end of the procedure.

Safety outcomes were defined as procedural complications (coronary perforation, slow or no reflow, new coronary thrombus, ventricular arrhythmia, vessel occlusion, and device failure) and device failure (inability to cross the lesion, malfunction) along with the rate of major adverse cardiac events (MACCE), defined as cardiac death, MI, cerebrovascular events and TVR at in-hospital and 6-month follow-up. Clinical follow-up was conducted by personal appointment or telephone contact at regular intervals every six months after the index procedure. Data from future follow-ups will be provided as they become available.

### 2.5. Statistical Analysis

Descriptive data are presented as numbers and percentages for categorical variables and as mean with standard deviation for parametric variables and median with interquartile range for nonparametric variables, respectively, after assessing the normality of distribution and homogeneity of variance using the Levene test and the Shapiro–Wilk test, respectively, with a significance level of 0.05 chosen for all statistical analyses. For numerical variables, the number of non-missing values is presented as a percentage of the total cohort analyzed (n, (%)). Statistical analysis was performed by trained medical statisticians using R version 4.0.4.

## 3. Results

**Figure 2:**
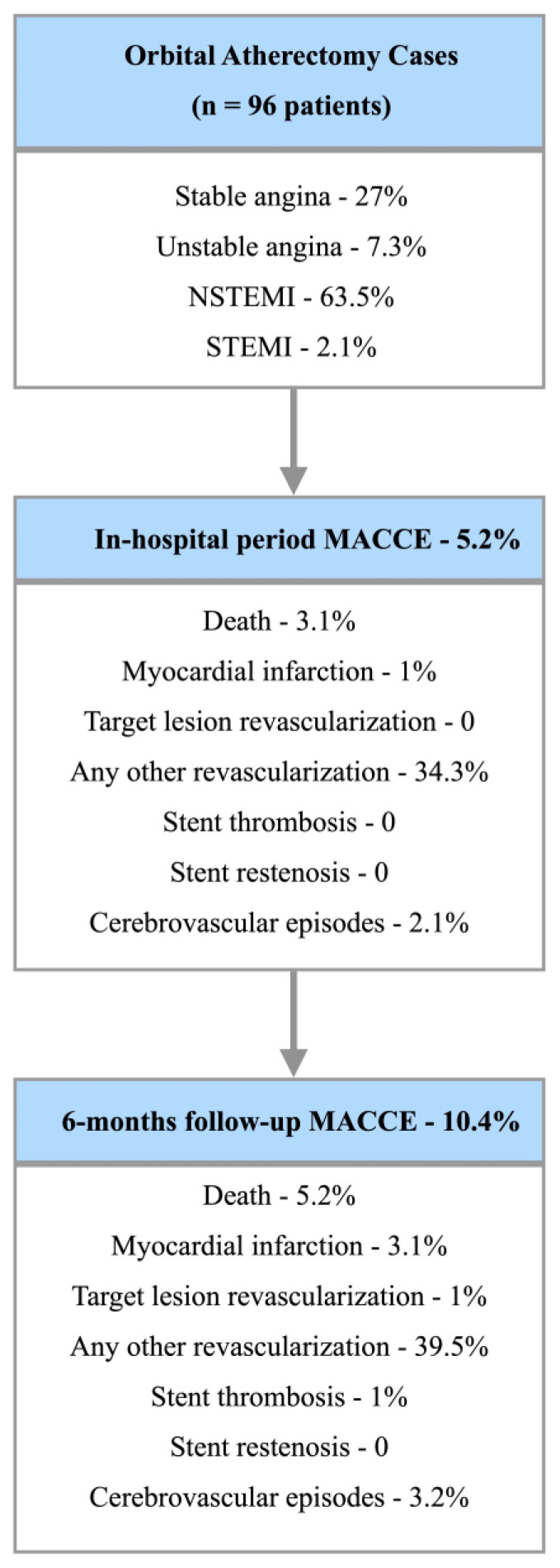
Study results and cohort characteristics.

The study evaluated 96 consecutive cases of orbital atherectomy performed at both cardiac centers, primarily in the ACS subset (73%). The study population was predominantly male (66.6%) with a mean age of 71.6 years. The study cohort was characterized by a high prevalence of cardiovascular risk factors including hypercholesterolemia (96.8%), hypertension (91.6%), chronic heart failure (48.9%), and diabetes mellitus (45.5%). Furthermore, more than one in three subjects had COPD, atrial fibrillation, a history of myocardial infarction, and/or had undergone previous revascularization. The high level of comorbidity was reflected in post-procedural pharmacotherapy: the vast majority of patients received acetylsalicylic acid (94.7%), statins (93.8%), angiotensin-converting enzyme inhibitors (ACEI) or angiotensin receptor blockers (92.7%), and b-blockers (90.6%). Notably, a relatively large proportion of patients (63.5%) received clopidogrel as part of DAPT. Table 1 shows the baseline clinical characteristics of the study population. Figure 2 presents the study flow chart and results.

Table 2 shows the baseline procedural characteristics of the study population. In the study cohort, the high anatomic complexity of CAD was observed—the Syntax score I reached a median of 22.5 (15.8–25) with a subsequent Syntax II-PCI score of 41.9 (12.6) and an estimated 4-year mortality of 24.5% (9.1–32.6). Therefore, we observed a high prevalence (34.3%) of “any other revascularization” during the index hospitalization. This was mainly related to the high number of PCIs performed as planned staged revascularization to complete PCI of other no-culprit lesions. Nevertheless, we observed a relatively low number of periprocedural complications—only two cases of slow-flow phenomena and one vessel perforation. In the vast majority (75.6%) of orbital atherectomy-PCI procedures, the device was used as the initial debulking tool. In nearly one in three cases, the OA was used due to unsuccessful initial lesion predilatation with an NC balloon. In 12.5% of cases, OA was used due to the inability to cross a lesion with the balloon catheter. The clinical success rate in this highly challenging lesion (total DES length per procedure—57.7 mm (39–72)) was high, reaching 92.7%. In the remaining seven cases after the OA procedure, we were still unable to achieve full expansion of the NC balloon catheter (at least 20% of under expansion; with at least 16 atm.) during post-dilatation, and prior to stent implantation, we were forced to use additional lesion preparation with shock wave intravascular lithotripsy (S-IVL). In the study cohort, we observed a relatively high prevalence of radial access (89.6%). This was supported by the high use of 6F therapeutic catheters (79.1%). In all cases studied, operators used the low-speed mode. In 57.2% of cases, the high-speed mode was additionally used.

During the in-hospital period, we observed a MACCE of 5.2%. Two deaths occurred during the initial hospitalization. One was a patient with multiple comorbidities and advanced CAD (syntax score 31) who died 4 days after LM/LAD -OA-PCI. Death occurred without prodromal symptoms of sudden cardiac arrest (PEA) despite prolonged resuscitation. The second fatal in-hospital case was a patient with advanced heart failure and a giant aortic aneurysm (compressing the lung causing advanced respiratory failure) who died from multiple organ dysfunction. In both fatal cases, the patients were consulted about possible cardiac surgery prior to PCI and were disqualified due to unacceptably high surgical risk. In addition, we observed one episode of periprocedural TIA and one stroke 3 days after the index procedure. Regarding in-hospital MACCE, we additionally observed one episode of periprocedural MI. The event was related to acute side branch (Cx) occlusion following target lesion (LM/LAD) stenting.

At the 6-month follow-up, MACCE (10.4%) was reported with only one target lesion failure—fatal subacute (14 days after the initial procedure) LAD stent thrombosis. Two additional patients died during this observation period. One subject with high comorbidity and advanced life-limiting conditions died of multi-organ dysfunction approximately 3 months after the index procedure; a second death was observed in a subject with a low ejection fraction scheduled for ICD implantation who experienced a fatal episode of exacerbation of advanced heart failure approximately 3 months after discharge. In addition, one case of non-TLR-related NSTEMI was reported in the study cohort. Furthermore, one subject experienced a TIA 15 days after discharge. All data regarding clinical follow-up were pooled in Table 3.

## 4. Discussion

The prevalence of severely calcified lesions treated with percutaneous coronary intervention is rapidly increasing [19,20]. Severely calcified lesions undoubtedly increase the complexity of PCI, often requiring the use of a wide range of devices and sophisticated techniques to achieve clinical success [21,22]. Adequate plaque modification before stenting is critical to procedural and long-term PCI success, as calcifications often preclude adequate stent expansion. Classic balloon-dependent lesion preparation methods often prove inadequate in highly calcified lesions [23,24]. Therefore, several dedicated devices have been developed to achieve proper plaque modification. Before orbital atherectomy and ShockWave intravascular lithotripsy entered clinical practice, rotational atherectomy was the most commonly used advanced debulking device [25,26]. While the safety and efficacy of RA have been well documented [27,28], data on OA are still scarce and mostly derived from single-arm, strictly controlled studies [29,30,31,32] designed to obtain approval for the use of a device in clinical practice.

Our study is one of the first presenting “real-life” data from the Lower-Silesia Orbital Atherectomy Registry (LOAR), which evaluates the mid-term outcomes of percutaneous coronary intervention-assisted orbital atherectomy device use in an all-comer cohort. In our high-risk cohort, 73% of subjects had ACS, with a Syntax I score of (22.5 (15.8–25) and mean Syntax II PCI score of 41.9 ± 12.6). Although data on the lesion complexity (syntax score) are not available in Orbit I and II trials, data on the lesion length (57.7 (39–72) mm vs. 13.4 ± 4.5 mm and 18.9 ± 0.4 mm) and number of DES implanted during the procedure (1.8 ± 0.4 vs. 1.1 ± 0.3 and 1.3 ± 0.0) suggest a higher lesion complexity in our study cohort. Of note, patients with ACS were excluded from the Orbit I and II trials. Despite these differences, we achieved comparable angiographic outcomes (92.6% vs. 93.4% and 88.9%) (defined as final residual stenosis after OA of less than 20% vessel diameter).

Data regarding clinical outcomes are also encouraging as the results of our study suggest a similar rate of short-term TLR (1% vs. 0% and 0.7%) and MACE (5.2% vs. 4% and 9.8%). An equal relationship was observed in terms of mid-term outcomes TLR (1% vs. 2% and 4.7%) and MACE (10.4% vs. 8% and 16.4%) [32,33]. Although pharmacotherapy, particularly with regard to dual antiplatelet therapy (DAPT), was not fully in line with the latest ESC/ESH guidelines [34], it must be emphasized that our data come from a real-life registry, and in the general population, deviations from the latest recommendations for antiplatelet therapy are common findings [35,36,37]. Similar results were obtained in other small-number trials [38,39,40,41,42,43]. The results obtained suggest a relatively low rate of adverse outcomes compared to previously published data on alternative treatment strategies. In this regard, analysis of the performance of rotational atherectomy in similar subpopulations of patients in the ROTAXUS and PREPARE-CALC trials revealed significantly higher rates of short- and medium-term TLR and major adverse cardiac events [44]. Furthermore, the ROTAXUS trial failed to demonstrate any clinical benefit of rotational atherectomy compared with standard balloon predilatation [45]. Although data comparing RA and OA are scarce [40,46,47], juxtaposing them with the results obtained in our study suggests that OA may have some clinical advantage over RA. This fact may be related to the more favorable mechanism of action of OA—the ability to provide bidirectional passage with additional orbital movement inside the lumen vessel, allowing interaction with deep calcium deposits, reducing the size of debris generated during the ablation process, and providing continuous blood flow through the vessel during the procedure [27]. If we compare the results obtained in our study with a similar “real-life” registry that refers to the alternative, novel debulking device—the S-IVL—we can see a similar level of short and mid-term TLR and MACE [48,49,50]. However, it is important to remember that the two devices appear to be designed for slightly different plaque morphologies based on their mechanism of action [51,52,53] (OA is more suitable for long, diffuse, tight lesions; S-IVL is more appropriate for short, focal, or profound calcifications). Therefore, the combination of different debulking methods may be useful to treat extreme-calcification-resistant lesions [21,22]. Our registry also demonstrates the benefits of combining both therapeutic methods. In seven cases, OA was insufficient for adequate lesion preparation. In these extremely calcified cases, we were forced to use additional support of the S-IVL after the successful passage of the crown due to an initial under-expansion of the NC catheter.

What needs to be emphasized is that the orbital atherectomy device turned out to be a relatively safe procedure in our highly demanding study cohort. We noticed only one episode of vessel perforation and two episodes of slow flow phenomena. Several factors have an impact on the procedure safety. A relatively flat learning curve was observed with the less demanding technical features of the procedure compared to traditional RA (ability to bi-directional burr passage reduce the probability of burr entrapment; single crown without necessary of burr escalation, good trackability of ViperWire; a predestined lubricant cocktail that reduces thermal injury and the likelihood of a slow-flow phenomenon). Additionally, due to the small size of the burr, the vast majority of procedures in our study cohort were performed by radial access and the standard size of the guide catheter (6F), which, in combination with a relatively high prevalence of intravascular imaging in the study cohort (procedures from everyday practice with a high percentage ACS setup), might have a strong impact on the procedure’s safety and efficiency [54,55,56,57].

Despite the aforementioned good tractability of ViperWire, different tactics can be used to address lesions with ViperWire in OA procedures. It is important to note that in our real-world study, we did not have a standardized protocol for OA, and the “wiring strategy” varied slightly from operator to operator. However, in most cases, the treated vessel was pre-wired with the standard “workhorse” wire, and then the ViperWire was advanced to the vessel and navigated with the previously placed wire. If we are unable to cross the lesion with the ViperWire, we exchange it with the microcatheter (via a preciously placed wire). After a successful OA procedure, the vessel was rewired with the workhorse wire (or exchanged with the microcatheter) and PCI was performed with the workhorse wire. Rewiring to the workhorse wire was mainly dictated by the poor support provided by ViperWire during stent delivery. In cases where OA was performed as a bail-out strategy, mainly due to the presence of uncrossable lesions, the ViperWire was placed in the distal part of the vessel via the microcatheter.

Similarly, the OA characteristics (number of runs, speed, and range) were not standardized in our study cohort and were left to the operator’s discretion. The main determinants in the decision-making process were the resistance generated during the crown crossing, the sound phenomenon accompanying the burr passage, and, partially, the angiographic appearance of the lesion. Therefore, in our opinion, there is a strong need for randomized studies focused on the evaluation of different OA performance strategies to apply the most convincing and adaptable technique depending on the initial lesion characteristics.

## 5. Limitations

Our study has several limitations. The first is the non-randomized retrospective study design with the lack of a control group. The second is the lack of external core laboratory analysis. Furthermore, the pharmacotherapy used in the study cohort, particularly regarding dual antiplatelet therapy (DAPT), was not fully consistent with the most recent ESC/ESH guidelines. In addition, a high level of comorbidity in the study cohort may have influenced the results obtained. Finally, the additional use of a debulking device (S-IVL) in the most demanding case may complicate the analysis of the study results, but we have to remember that the vast majority of cases were performed in the ACS subset, where the optimal procedural outcome is crucial for patient survival and should be achieved with all available tools and techniques.

## 6. Conclusions

Mid-term (6-month) data from the Lower Silesia Orbital Atherectomy Registry (LOAR) suggest the good efficacy and safety profile of orbital atherectomy in a high-risk all-comers cohort with calcified lesions. Despite the favorable results, large randomized trials, especially in comparison with other advanced plaque modification techniques, are needed to determine the optimal treatment for patients with severely calcified CAD.

## Figures and Tables

**Table 1:** Baseline clinical characteristic of the study cohort.

Orbital Atherectomy N-96	
Clinical Features	
Age, mean (SD)	71.6 (7.9)
Gender male, n (%)	64 (66.6)
Stable angina, n (%)	26 (27)
Unstable angina, n (%)	7 (7.3)
NSTEMI, n (%)	61 (63.5)
STEMI, n (%)	2 (2.1)
Non-diabetic hyperglycemia, n (%)	13 (13.5)
Diabetes mellitus, n (%)	44 (45.5)
Chronic heart failure, n (%)	47 (48.9)
Hypertension, n (%)	88 (91.6)
Hyperlipidemia, n (%)	93 (96.8)
Atrial Fibrillation, n (%)	33 (34.3)
History of PCI, n (%)	38 (39.5)
History of MI, n (%)	37 (38.5)
History of CABG, n (%)	9 (9.4)
COPD, n (%)	32 (33.3)
History of stroke, n (%)	11 (8.4)
Moderate/severe valvular heart disease, n (%)	18 (18.7)
Chronic kidney disease, n (%)	23 (23.9)
LVEF (%), mean (SD)	47.8 (12.7)
Creatinine level (µmol/L), median (Q1–Q3)	85.1 (68.2–93)
Post-procedural Pharmacotherapy	
Acetylsalicylic Acid, n (%)	91 (94.7)
Clopidogrel, n (%)	61 (63.5)
Ticagrelor, n (%)	20 (20.8)
Prasugrel, n (%)	15 (15.6)
Statins, n (%)	90 (93.8)
NOAC/VKA, n (%)	34 (35.4)
ACEI/ARB, n (%)	89 (92.7)
B-blocker, n (%)	87 (90.6)
CCB, n (%)	43 (44.7)
Oral antidiabetic, n (%)	49 (51.0)
Insulin, n (%)	10 (10.4)

Abbreviations: SD—standard deviation; STEMI—ST-elevation myocardial infraction; NSTEMI—no ST-elevation myocardial infraction; COPD—chronic obstructive pulmonary disease; LVEF—left ventricular ejection fraction; PCI—percutaneous coronary intervention; CABG—coronary artery bypass grafting; ACEI—angiotensin-converting-enzyme inhibitors; ARB—angiotensin receptor blockers; NOAC—non-vitamin K antagonist oral anticoagulants; VKA—vitamin K antagonists; B-blocker—beta blocker; CCB—calcium channel blocker.

**Table 2:** Baseline procedural characteristics of the study cohort.

Orbital Atherectomy N-96	
Vessel treated:	
LM, n (%)	29 (30.2)
LAD, n (%)	37 (38.5)
LCX, n (%)	9 (9.4)
RCA, n (%)	21 (21.8)
Syntax I score, median (Q1–Q3)	22.5 (15.8–25)
Syntax II—PCI score, mean (SD)	41.9 (12.6)
Syntax II PCI four-year mortality, median (Q1–Q3)	24.5 (9.1–32.6)
Syntax II—CABG score, mean (SD)	39.2 (10.7)
Syntax II CABG year mortality, median (Q1–Q3)	19.3 (8.2–28.3)
Primary orbital atherectomy procedure, n (%)	65 (75.6)
Unsuccessful predilatation, n (%)	32 (33.4)
Uncrossable lesion	12 (12.5)
CTO lesions, n (%)	4 (4.6)
Post atherectomy S-IVL use, n (%)	7 (7.3)
Reference vessel diameter (RVD) (mm), mean (SD)	3.1 (0.5)
Initial stenosis diameter (%), mean (SD)	86.1 (7.4)
Final stenosis diameter (%), mean (SD)	8.1 (3.1)
Low-speed OA use, n (%)	96 (100)
High-speed OA use, n (%)	55 (57.2)
OA duration time (s), mean (SD)	246.7 (86.5)
Postdilatation, n (%)	91 (94.7)
Post-dilatation pressure (atm), mean (SD)	18.6 (2.5)
Number of DES per procedure, mean (SD)	1.8 (0.4)
Total DES length per procedure (mm), median (Q1–Q3)	57.7 (39–72)
Intravascular guidance, n (%)	52 (54.1)
Clinical success, n (%)	89 (92.7)
Slow-flow phenomena, n (%)	2 (2)
Vessel perforations, n (%)	1 (1)
Radial access, n (%)	86 (89.6)
6F guide catheter, n (%)	76 (79.1)
7F or larger guide catheter, n (%)	20 (23.2)
Radiation dose (mGy), n (%), median (Q1–Q3)	1442.8 (792.2–1921.0)
Contrast volume, n (%), median (Q1–Q3)	258.2 (157.7–300.0)

Abbreviations: SD—standard deviation; PCI—percutaneous coronary intervention; LM—left main; LAD—left anterior descending; Cx—circumflex artery; RCA—right coronary artery; DES—drug-eluting stent; DEB—drug eluting balloon; S-IVL—shockwave intravascular lithotripsy; CTO—chronic total occlusion; CABG—coronary artery bypass grafting; MACCE—major adverse cardiac and cerebrovascular event.

**Table 3:** Clinical outcomes.

Orbital Atherectomy N-96	
In-hospital period	
MACCE, n (%)	5 (5.2)
Death, n (%)	2 (3.1)
Myocardial infarction, n (%)	1 (1.0)
Target lesion revascularization, n (%)	0 (0)
Any other revascularization, n (%)	33 (34.3)
Stent thrombosis, n (%)	0 (0)
Stent restenosis, n (%)	0 (0)
Cerebrovascular episodes, n (%)	2 (2.1)
6-month follow-up	
MACCE, n (%)	10 (10.4)
Death, n (%)	5 (5.2)
Myocardial infarction, n (%)	3 (3.1)
Target lesion revascularization, n (%)	1 (1.0)
Any other revascularization, n (%)	38 (39.5)
Stent thrombosis, n (%)	1 (1.0)
Stent restenosis, n (%)	0 (0)
Cerebrovascular episodes, n (%)	3 (3.2)

Abbreviations: MACCE—major adverse cardiac and cerebrovascular event.

## Data Availability

All data not included in the manuscript are available after contacting the corresponding author in accordance with local legal regulations.
